# Multiple paternity is a shared reproductive strategy in the live-bearing surfperches (Embiotocidae) that may be associated with female fitness

**DOI:** 10.1002/ece3.1071

**Published:** 2014-05-12

**Authors:** John R LaBrecque, Yvette R Alva-Campbell, Sophie Archambeault, Karen D Crow

**Affiliations:** Department of Biology, San Francisco State University1600 Holloway Ave., San Francisco, California, 94132

**Keywords:** Bateman gradient, *Cymatogaster*, *Hyperprosopon*, multiple paternity, reproductive strategy, surfperch

## Abstract

According to Bateman's principle, female fecundity is limited relative to males, setting the expectation that males should be promiscuous, while females should be choosy and select fewer mates. However, several surfperches (Embiotocidae) exhibit multiple paternity within broods indicating that females mate with multiple males throughout the mating season. Previous studies found no correlation between mating success and reproductive success (i.e., a Bateman gradient). However, by including samples from a broader range of reproductive size classes, we found evidence of a Bateman gradient in two surfperch species from distinct embiotocid clades. Using microsatellite analyses, we found that 100% of the spotfin surfperch families sampled exhibit multiple paternity (Hyperprosopon anale, the basal taxon from the only clade that has not previously been investigated) indicating that this tactic is a shared reproductive strategy among surfperches. Further, we detected evidence for a Bateman gradient in *H. anale*; however, this result was not significant after correction for biases. Similarly, we found evidence for multiple paternity in 83% of the shiner surfperch families (*Cymatogaster aggregata*) sampled. When we combine these data with a previous study on the same species, representing a larger range of reproductive size classes and associated brood sizes, we detect a Bateman gradient in shiner surfperch for the first time that remains significant after several conservative tests for bias correction. These results indicate that sexual selection is likely complex in this system, with the potential for conflicting optima between sexes, and imply a positive shift in fertility (i.e., increasing number) and reproductive tactic with respect to the mating system and number of sires throughout the reproductive life history of females. We argue that the complex reproductive natural history of surfperches is characterized by several traits that may be associated with cryptic female choice, including protracted oogenesis, uterine sac complexity, and sperm storage.

## Introduction

According to Bateman's principle, female fecundity is limited relative to males; therefore, reproductive success is expected to increase with multiple mates in males but not in females (Bateman 1948). This difference in sexual selection between males and females is referred to as a “Bateman gradient” or “sexual selection gradient” (Kvarnemo and Simmons 2013) and remains contentious in the literature for a variety of reasons, including difficulty in estimating mating success in nature as a direct indicator of fitness (Arnold and Duvall 1994). Here, we investigate a unique system, which allows for the most accurate estimation of mating success (number of inferred sires) and reproductive success (number of offspring) in a natural setting because, unlike most fishes, pregnant females can be collected for the quantification of brood size and multiple paternity. Surfperches (Embiotocidae) exhibit internal fertilization, a protracted gestation period, and give birth to relatively few (usually <100) well-developed offspring. In some species, the juveniles are sexually mature at birth (Wiebe 1968; Warner and Harlan 1982; Schultz 2008), representing a tactic that could be considered the ultimate parental investment strategy. Because female investment is so high, with internal development to the subadult stage, females should be highly discriminating and choose few mates, but this is not what has been observed in natural populations. Multiple paternity has been documented in three genera of surfperches (Reisser et al. 2009; Liu and Avise 2011, 2012) implying that females may benefit from multiple mating. However, these studies have not found a correlation between brood size and number of sires (i.e., a Bateman Gradient).

Currently, it is unclear whether multiple paternity is a shared reproductive strategy by all members of the Embiotocidae. The family consists of two subfamilies: the Embiotocinae and the Amphistichinae; however, multiple paternity has only been shown for four species within the Embiotocinae. *Embiotoca jacksoni,* and *E. lateralis* were characterized as having two to nine sires per brood (Reisser et al. 2009); the shiner surfperch, *Cymatogaster aggregata,* was characterized as having one to eight sires per brood (Liu and Avise 2011); and the only freshwater surfperch, *Hysterocarpus traski*, has one to four sires per brood (Liu and Avise 2012). According to the phylogeny proposed by Bernardi and Bucciarelli (1999), these species represent taxa within the subfamily Embiotocinae. However, no member of the sister taxon, the Amphistichinae, has previously been evaluated. Interestingly, these natural groups exhibit morphological differences in reproductive anatomy that could be associated with differences in sexual strategies. While all surfperches are internal fertilizers, members of the Embiotocinae exhibit a “penis-like flask organ” that has been described as an enlarged swelling in the anterior anal fin with a “mammary protuberance” (Blake 1868). In contrast, members of the Amphistichinae have hook-like, bony plates with serrated edges in the middle of the anal fin (Tarp 1952). These anal fin modifications develop before parturition in some species (Schultz 1990) and are clearly important in mating. However, the functional significance of the observed disparity in reproductive anatomy or mating strategies between the Amphistichinae and Embiotocinae has not been addressed. It is therefore essential to evaluate a member of the Amphistichinae to understand the evolution of reproductive strategies among the surfperches.

**Figure 1:**
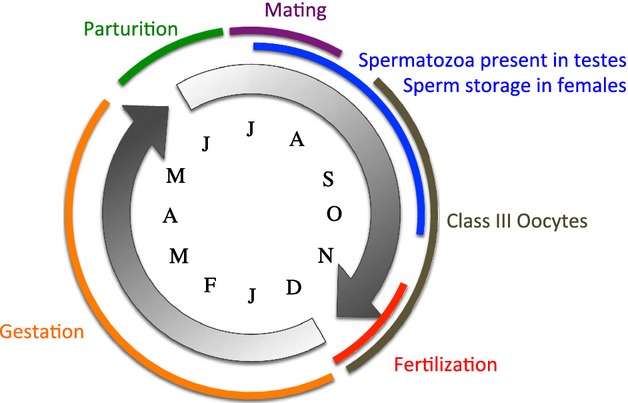
Annual reproductive cycle for *Cymatogaster aggregata,* after Wiebe 1968. Letters indicate the month of the year. Note that males are only reproductive for 4 months out of the year (July–October), while females are engaged in aspects of reproduction throughout the year. Importantly, oogenesis continues to occur well after mating and spermatogenesis subsist.

Several aspects of the reproductive cycle of female surfperches, including protracted ovulation and uterine sac complexity, suggest mechanisms for cryptic female choice and sexual selection. Because this represents a potential mechanism for sexual selection on males, we became interested in investigating whether a female Bateman gradient exists in surfperches, and why this pattern may have gone undetected in previous studies. The reproductive cycle is best characterized for shiner surfperch, *Cymatogaster aggregata*, Fig. 1, but may vary slightly in other species (Froeshke et al. 2007). In *C. aggregata*, mating occurs primarily in July and August (Wiebe 1968), but mature sperm are present in males for two more months, through October. Importantly, oocyte development continues for approximately two months after mature sperm are spent in males, and fertilization is delayed until November to December, well after the mating season, indicating that females store sperm for up to five months. During this time, the sperm is tightly associated with sheets of tissue inside the uterus (Gardiner 1978), an unpaired structure referred to as the “ovary” and “uterus” interchangeably in the literature. These sheets form complex structures (Behrens 1977) and likely facilitate the multiple reproductive roles of the uterus, including sperm storage, oocyte maturation, fertilization, and gestation. During the 5–6-month gestation period, embryos absorb nutritive materials and oxygen directly from the mother through ovarian fluid and vascularization of embryonic fins and ovarian sheets (Wiebe 1968; Behrens 1977). Due to their relatively high investment, the expectation is that females should exhibit strong sexual selection on males.

The objectives of this study are to:

1. Evaluate whether multiple paternity is a shared reproductive tactic of Embiotocids by inferring patterns in parentage for *Hyperprosopon anale*-the basal Amphisticine taxon.
2. Evaluate whether multiple paternity has arisen in association with a sexual selection gradient (i.e., a Bateman gradient) in two surfperch species.
3. Characterize uterine complexity and test for paternal skew to determine whether these traits are correlated in *Cymatogaster aggregata*.

## Materials and Methods

### Sampling and paternity analyses of surfperch families inferred from microsatellites

Twenty-four gravid *Hyperprosopon anale* females were collected off Pacifica, CA, during May 2011 using hook and line and 23 *Cymatogaster aggregata* females were collected using a beach seine at Princeton, CA in June 2010. Fin clips from mothers and all offspring were preserved in ethanol for DNA extraction. Brood size and standard length were quantified for all gravid females. The uterine sacs of 10 *C. aggregata* were preserved intact and investigated for patterns in paternal skew. Multiple paternity was assessed via microsatellite analysis of six loci developed for surfperches of the genus *Embiotoca* by Reisser et al. (2009), and six loci developed for *Cymatogaster aggregata* by Liu and Avise (2011), (Table 1). Loci were optimized for *Hyperprosopon*, with the following initial PCR conditions: 12.5 *μ*L Jumpstart *Taq* polymerase, 200 *μ*mol/L, 0.25 *μ*L labeled M13 forward primer, 50 *μ*mol/L, 0.25 *μ*L labeled reverse primer, 50 *μ*mol/L, 1 *μ*L extracted DNA, and 11 *μ*L water. Thermal cycling parameters for amplifications were as follows: 95°C for 3 min, then 35 cycles each at 95°C for 20 sec, 60°C for 20 sec, and 72°C for 30 sec, followed by one cycle of final elongation at72°C for 10 min. To visualize microsatellite variation, we employed a modified PCR approach utilizing a specific primer modified with 5' M13 sequence and a FAM labeled M13 primer cocktail according to Schuelke (2000). Allele scoring was performed using GENESCAN 2.1 (version 2.1, Applied Biosystems, Foster City, CA) and GENEIOUS 6.0 (Geneious [Bibr b8000]). Paternity analyses were performed using the software COLONY 2.0 (Wang 2004; Wang and Santure 2009), which implements a maximum-likelihood framework to evaluate parentage and sibling relationships within broods, taking maternal information into account as well as estimating allele frequencies. Paternal contribution was assessed for reproductive skew with the program SKEW CALCULATOR 2003 (Nonacs 2003) using 10,000 permutations. Correlations between standard length versus brood size and brood size versus number of sires were evaluated by linear regression implemented in the software R version 2.15 (R Development Core Team 2010).

**Table 1:** Characteristics of 13 microsatellite loci used for this study. Six were developed for *Cymatogaster* (Liu and Avise 2011) and seven were developed for *Embiotoca* (Reisser et al. 2009). Data from previous studies are presented in the middle (CagS, top; and Ela and Eja, below). Data produced in this study are displayed on the right for CagC and Han. Data Exclusion probabilities reflect condition when one parent is unknown, *n* equals the number of alleles present for each locus

		Liu & Avise 2012						This study									
			
Locus	Primers	Repeat	Range CagS	nCagS	Exclusion probability	He	Ho	Range CagC	nCagC	Exclusion probability	He	Ho	Range Han	nHan	Exclusion probability	He	Ho
cag15	*F-TGTAAAACGACGGTTAGGATTCAAAA R-ATTAGAGCAGGGTAGGATTA	(AC) 25	205–263	22	0.875	0.9556	1	206–272	26	0.837	0.9371	0.875	N/A	N/A			
cag27	*F-TGTAAAACGACGGTAAACAGCAGGAT R-GAAGGAGATGAAAGGAACAA	(GT) 27	218–288	22	0.816	0.9334	0.954	211–291	26	0.818	0.9176	0.875	195–248	2			
cag28	*F-TGTAAAACGACGGTGTTTAGACAGAA R-ACAAATGGCAGTGAAAGGAG	(AC) 19	251–292	22	0.870	0.9524	1	236–302	25	0.862	0.9273	0.9167	246–364	45	0.904	0.9739	0.913
cag29	*F-TGTAAAACGACGGCACCTGTCTCAAC R-GAACTAACTCTTCCAGCAAA	(AC) 35	166–258	28	0.906	0.9672	1	161–249	30	0.804	0.9264	0.9583	165–193	11	0.595	0.7952	0.6087
cag36	*F-TGTAAAACGACGGGGCATGTGATGAG R-GCCTGGCSGSTGTGAAAGAG	(GT) 20	104–142	15	0.794	0.9102	1	104–144	17	0.793	0.7917	0.7917	80	1			
cag50	*F-TGTAAAACGACGGACGGAAATGTGAT R-ACCTGCTTCTTTCAGGGACA	(GT) 36	123–217	26	0.890	0.962	1	136–218	28	0.857	0.9548	1	120	1			
All loci										0.999							
			Reisser et al. 2009														
																
			nEla	nEja													
EJ_A2	F-AGCAAAGGTCAAAGGTCAA R-TTGTGGCTGTTGTTTATGG	(CA) 20	14	6									94–254	6	0.2981	0.5333	0.2609
EJ_A5	F-AACCGCTGAGTAAGTAAACATC R-TCATCCCCATCATATTTATAGC	(CA) 30	12	15									N/A				
EJ_A11	F-ACTTCCATGACAACAAAGTAGG R-CAAAATAAGCCAAGTGTGATG	(CA) 24	18	7									226	1			
EJ_A12	F-GAAAGAAGCTCAATGCAATCAC R-AGCAGCTCTCAGATCAGAGGTA	(CA) 24	N/A	5									92–174	2			
EJ_B1	F-ACTCGGACAGTAAAGCTGAGG R-AAAATGTCTCCTTGCAGGATC	(GATG) 14	5	N/A									104	1			
EJ_B5	F-CCACCTGGGGCTAAACTG R-CACGGCAGACAGAGCAAC	(CATC) 15	N/A	8									92–184	3			
EJ–B8	F-GGTCGTATTTTGCAGTATGC	(GATG) 30	N/A	7									N/A				

* Indicates M13 reverse tail (GGAAACAGCTATGACCATG-30) labeled with Fam, Hex, or Ned.

## Results

### Multiple paternity and Bateman gradient in *Hyperprosopon anale –* the basal Amphisticine taxon

**Figure 2:**
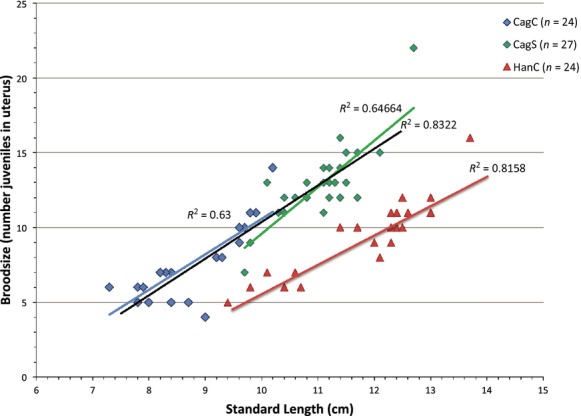
Standard length versus brood size in two species of surfperch. Diamonds represent two populations of *Cymatogaster aggregata* (Cag), one from Central Claifornia (CagC, blue), and the other from southern California (CagS, green). Black trend line indicates relationship for combined populations. Red triangles represent *Hyperproson anale* sampled from Central California

**Figure 3:**
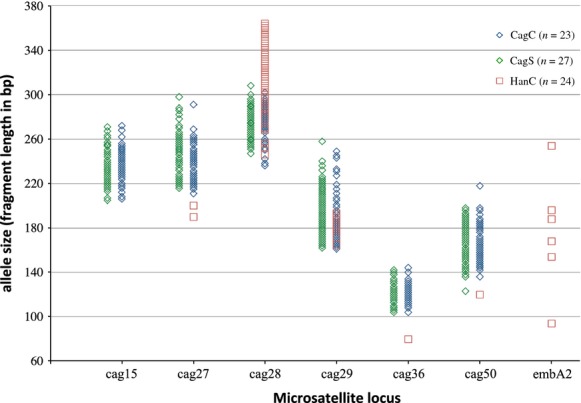
Variation in allele sizes for seven microsatellite loci used for paternity assignments in two surfperch species. Variable loci for *Cymatogaster aggregata* are represented by blue and green diamonds (blue = CagC, Central California, this study; green = CagS, southern California, from Liu and Avise 2011). Red squares represent allelic variation in *Hyperprosopon anale* (HanC*,* central California).

**Figure 4:**
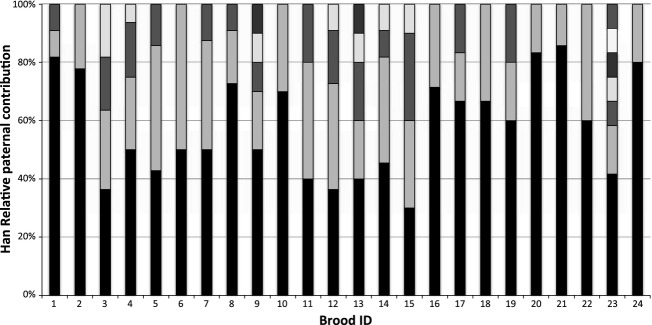
Relative paternal contributions to *Hyperproson anale* brood. Assignment of paternity within broods, for *Hyperprosopon anale*. Each bar represents all offspring within a brood from a single female. Shading indicates the number of offspring attributed to genetically distinct sires.

**Figure 5:**
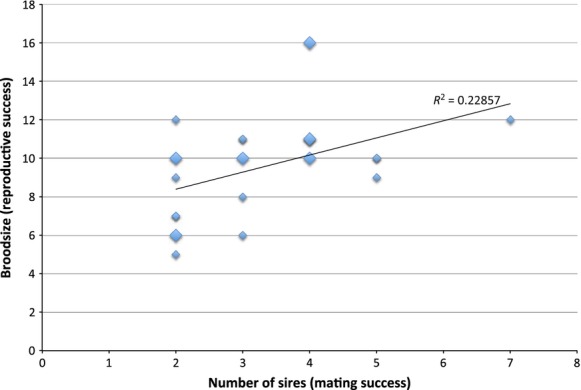
Bateman gradient inferred from 24 pregnant females of spotfin surfperch (*Hyperprosopon anale*). Mating success (number of sires) versus reproductive success (brood size) in 24 pregnant *H. anale* females, inferring a female Bateman gradient. This positive correlation is no longer significant when corrections are applied to control for variation in brood size. Larger diamonds represent multiple data points at that locus.

Twenty-four families of spotfin surfperch (*Hyperprosopon anale*) were evaluated with brood sizes ranging from 5 to 16, and a total of 228 offspring. Pregnant females ranged in size from 94–137 mm SL, and there was a clear correlation between female standard length and brood size (*P* < 0.001, Table 3, Fig. 2). Of the 13 microsatellite loci utilized, 10 were successfully amplified in *H. anale*, but only three were variable (Fig. 3, Table 1). All three were informative for paternity assignment, but only one exhibited extremely high variability that would be considered optimal, with allele frequencies in Hardy–Weinberg equilibrium (cag28, *n* = 24 moms, *P* = 0.2297). Due to the few tractable markers for *H. anale*, and limited variability in two, it is likely that the estimated number of sires per brood represents a minimum. Even so, multiple paternity was inferred in every family evaluated, with an average of 2–7 sires per family (Fig. 4, Table 2). This level of multiple paternity is comparable with patterns found in other surfperch species, but we note the possibility that some males may have mated successfully without siring offspring. Because the number of sires is likely underestimated, we cannot make inferences about potential differences in reproductive tactics between the Amphistichinae and the Embiotocinae. Regardless, these data indicate that multiple paternity is a reproductive strategy shared by both surfperch subfamilies and that it likely arose before their diversification. Even more interesting is the observation that these data infer a Bateman gradient in *H. anale* for the first time in surfperch (i.e., a positive correlation between mating success and reproductive success, (*P* = 0.018, Table 3, Fig. 5). Because there is a positive correlation between female SL and brood size, the power to detect multiple sires increases with brood size, introducing a potential statistical bias. To correct for this, we randomly sampled the same number of offspring per brood (using the random number generator at “http://random.org”) and plotted the newly inferred number of sires as the independent variable in the regression. When families were subsampled with five and six offspring each (Table 3), the Bateman gradient was no longer significant in *H. anale*. Because the resampling procedure limits the slope of the regression to the maximum number of sires equal to the selected brood size, the power to detect a Bateman gradient requires a large sample size that is representative of the full range of variation in the mating strategy.

**Table 2:** Multiple paternity and number of sires for 23 broods of shiner surfperch and 24 broods of spotfin surfperch sampled off central CA. Brood size (*n*), female standard length, number of sires, and the number of offspring sired by up to seven males, as well as binomial skew (index B) and corresponding *P* values are inferred using the program COLONY. Bold *P* values indicate significance

*Cymatogaster aggregata* Central California												

Family	Brood size (*n*)	Standard length (mm)	Number of series	Sire 1	Sire 2	Sire 3	Sire 4	Sire 5	Sire 6	Sire 7	*B* value	*P*
Cag1	14	102	4	10	2	1	1				0.237	**0.002**
Cag2	6	79	3	4	1	1					0.056	0.342
Cag3	11	99	2	8	3						0.058	0.218
Cag4	9	96	3	6	2	1					0.099	0.169
Cag5	11	98	3	8	2	1					0.176	**0.026**
Cag6	5	87	3	3	1	1					−0.027	0.630
Cag7	5	78	3	2	2	1					−0.107	1.000
Cag8	7	82	3	4	2	1					0.000	0.426
Cag9	7	83	1	7								
Cag1O	6	78	1	6								
Cag11	6	73	1	6								
Cag12	5	80	2	4	1						0.080	0.380
Cag13	6	79	2	5	1						0.139	0.215
Cag14	8	92	3	5	2	1					0.052	0.304
Cag15	7	84	3	3	2	2					−0.082	1.000
Cag16	7	83	2	5	2						0.020	0.455
Cag17	7	83	3	3	3	1					−0.041	0.708
Cag18	7	82	3	5	1	1					0.122	0.138
Cag19	8	93	3	4	2	2					−0.042	0.742
Cag20	10	96	4	5	2	2	1				0.015	0.433
Cag21	4	90	2	3	1						0.000	0.622
Cag22	5	84	2	3	2						−0.080	1.000
Cag23	7	82	2	5	2						0.020	0.456
*Hyperprosopon anale* Central California												
Han1	11	130	3	9	1	1					0.292	**0.005**
Han2	9	120	2	7	2						0.099	0.180
Han3	11	130	4	4	3	2	2				−0.046	0.913
Han4	16	137	4	3	4	8	1				0.055	0.109
HanS	9	123	3	3	5	1					0.025	0.325
Han6	6	107	2	3	3						−0.083	1.000
Han7	8	121	3	4	1	3					−0.010	0.549
Han8	11	124	3	1	8	2					0.176	**0.026**
Han9	10	117	5	1	5	2	1	1			0.040	0.193
Han10	10	124	2	7	3						0.030	0.345
Han11	10	114	3	4	4	2					−0.040	0.781
Han12	11	126	4	1	2	4	4				−0.012	0.465
Han13	10	117	5	4	1	2	1	2			−0.020	0.601
Han14	11	123	4	4	5	1	1				0.037	0.220
Han15	10	124	4	3	3	3	1				−0.045	0.853
Han16	7	106	2	2	5						0.020	0.450
Han17	6	98	3	4	1	1					0.056	0.347
Han18	6	104	2	2	4						−0.028	0.693
Han19	10	123	3	6	2	2					0.040	0.250
Han20	12	130	2	10	2						0.181	**0.037**
Han21	7	101	2	6	1						0.184	0.127
Han22	5	94	2	2	3						−0.080	1.000
Han23	12	125	7	1	2	5	1	1	1	1	0.022	0.218
Han24	10	125	2	8	2						0.130	0.113

**Table 3:** Statistics describing the relationship between Standard length versus brood size and mating success versus reproductive success (Bateman gradient) in *Hyperprosopon anale* (Han) and *Cymatogaster aggregata* (Cag). CagC refers to analyses of data from a Central California population (this study), and CagS refers to a southern California population (Liu and Avise 2011). “All data” refers to analysis of combined data indicating a significant Bateman gradient. “Random draw” refers to statistical analyses from randomly selected subsets of individuals within broods to correct for the increased probability of detecting multiple sires in larger broods. *F*-statistics from Liu and Avise were unavailable. *,**,*** indicate order of magnitude of *P*-value

Family ID	Trial	*n* (# families)	*R*²	*F*	df	*P*-value	sig

Female SL versus Brood size							
Han	This study	24	0.816	97.41	22	<0.001	***
CagS	Liu & Avise	27	0.65		26	<0.001	***
CagC	This study	23	0.627	35.28	21	<0.001	***
CagC + CagS	Data Combined	50	0.832	238.1	48	<0.001	***
Mating success versus Reproductive success (Bateman gradient)							
Han	This study	24	0.229	6.519	22	0.018	**
	Random 5	23	0.038	0.823	21	0.375	
	Random 6	22	0.058	1.231	20	0.281	
CagS	Liu & Avise	27	0.007		26	0.66	
CagC	This study	23	0.101	2.37	21	0.139	
	Random 4	22	<0.001	0.001	20	0.978	
	Random 4	22	−0.121	2.759	20	0.112	
	Random 5	22	0.001	0.022	20	0.884	
	Random 5	22	0.073	1.585	20	0.223	
CagC + CagS	This study	50	0.265	17.26	48	<0.001	***
	Random 6	45	0.123	6.047	43	0.018	**
	Random 6	45	0.147	7.432	43	0.009	***
	Random 6	45	0.289	17.03	43	<0.001	***
	Random 4	49	0.172	9.737	47	0.003	**
	Random 5	48	0.164	9.052	46	0.004	**

### A new sexual strategy is inferred for the shiner surfperch, Cymatogaster aggregata, when the sample size is increased

**Figure 6:**
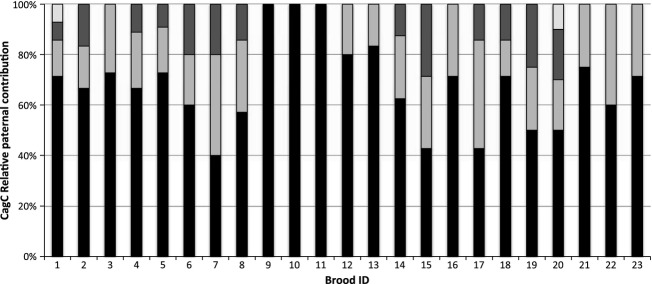
Relative paternal contributions to Central California *Cymatogaster aggregata* broods. Assignment of paternity within broods, for *Cymatogaster aggregata* sampled off Central California. Each bar represents all offspring within a brood from a single female. Shading indicates the proportion of offspring attributed to genetically distinct sires.

**Figure 7:**
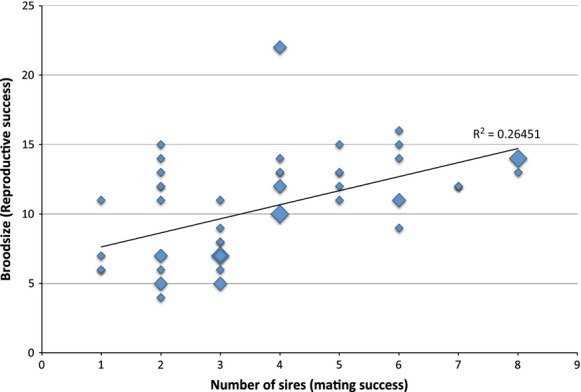
Bateman gradient inferred from 50 pregnant females of shiner surfperch (*Cymatogaster aggregata*). Mating success (number of sires) versus reproductive success (brood size) for 50 pregnant *C. aggregata* females, from combined data (this study, *n* = 23, CagC, and Liu and Avise 2011; *n* = 27, CagS) inferring a female Bateman gradient. Larger diamonds represent multiple data points at that locus. Positive correlation remains significant after correcting for biases.

A previous study found evidence for multiple paternity in the shiner surfperch, but no significant relationship between brood size and number of sires (i.e., Bateman gradient), likely due to small sample size and limited variation in female size. Liu and Avise (2011) evaluated 27 families sampled off southern California with brood sizes ranging from 7 to 22, with 348 offspring in total, and the expected significant correlation between female standard length and brood size (*P* = 0.66, Liu and Avise 2011). Using the same set of highly variable microsatellite markers developed for this species, we inferred multiple paternity in 20 of the 23 broods from pregnant females sampled off Central California with one to four sires per family, Fig. 6, Table 2, and the expected correlation between female standard length and brood size (*P* < 0.001, Table 3). Brood sizes ranged from 4 to 14 offspring, with 178 offspring in total. Similarly, we found no significant correlation between brood size and number of sires (i.e., Bateman gradient, *P* = 0.137, Table 3). However, we noticed that our study included smaller individuals ranging from 73 to 102 mm in standard length, while the Liu and Avise (*P* < 0.001, Liu and Avise 2011) study included larger individuals ranging from 97 to 127 mm standard length. Because detection of a Bateman gradient requires sampling across the range of variability existing in nature, particularly when statistical corrections are applied, it became clear that neither study had the sample size or statistical power to detect such a pattern. When we combined data sets from these two studies off California, we found evidence for a Bateman gradient that remains significant even after correcting for the correlation between female standard length and brood size (*P* < 0.001, Fig. 7, Table 3). This represents the first inference of a positive correlation between brood size and number of sires, or a Bateman gradient (*P* < 0.001, Fig. 7), in surfperch that remains significant after resampling for bias correction (Table 3). To standardize the number of offspring across all families examined, we randomly sampled six offspring using a random number generator and paternity was re-evaluated using the software COLONY (2009). This procedure was repeated three times, with six offspring randomly drawn, and again with five and four offspring randomly drawn, and evidence for a Bateman gradient remains significant in all trials (Table 3). We note that this is a conservative test because the slope of the regression is constrained by the number of offspring selected (i.e., always less that the actual brood size), and sampling across families is unequal because small families have fewer offspring excluded from the reanalysis than large families; therefore, we only included families with where the brood size was greater than the number drawn, so that each subsample had a random component.

### Combining samples from two populations of *Cymatogaster aggregata*

We include the following rationale for combining data from two different studies, with samples from central and southern California, because a reviewer was concerned that differences between these populations may indicate independent origins of reproductive strategies, or different factors structuring them. We did not feel this was a problem with respect to patterns of multiple paternity for several reasons. First, we demonstrate here that polyandry arose in the common ancestor of the surfperch family (with the inference of multiple paternity in the basal Amphisticine taxon), indicating that the origin of this shared reproductive tactic is not a population level trait. Second, it was fortuitous that our samples complemented the range of female standard lengths in the Liu and Avise study, with overlapping samples and similar slopes, and therefore, it is unlikely that these populations exhibit significant differences in body length. For example, the large females sampled off southern California were obviously once smaller and younger, with smaller broods given the observed correlation between brood size and female standard length that is well documented in surfperches (Baltz 1984). This is illustrated in Fig. 2 where the slope of the regression for brood size on female standard length is nearly identical, indicating a continuous trend. Additionally, we used the same primer sets as Liu and Avise that were developed for this species, and the allelic variation was comparably high in both populations, and therefore, our ability to detect the number of sires was equivalent. For thoroughness, we evaluated the connectivity between these two *C. aggregata* populations relative to other surfperch populations, by estimating genetic population structure using AMOVA for 50 females (23 individuals from the Central and 27 individuals from the southern population) based on the same microsatellite loci. Deviations from Hardy–Weinberg equilibrium and linkage equilibrium were estimated using ARLEQUIN 3.5.1.2 (Excoffier and Lischer 2010). The presence of null alleles was evaluated using the Oosterhout statistic in MICRO-CHECKER 2.2.3 (Van Oosterhout et al. 2004), and *F*-statistics were estimated with ARLEQUIN 3.5.1.2 (Excoffier and Lischer 2010). All loci exhibited null allele/dropout rates of 10% or less and were in Hardy–Weinberg equilibrium. We found a relatively low level of genetic structure between the two populations (*F*st = 0.044, *P* < 0.000) suggesting limited migration. And because surfperch lack a larval dispersal phase, and recruit directly to the adult habitat at birth, a significant level of population structure is not surprising. But remarkably, the observed level of genetic subdivision was still less than expected relative to other surfperch populations compared at the same geographic scale. For example, Bernardi (2000) found pairwise *F*st values based on mtDNA sequences that were 16 times greater in black surfperch (*Embiotoca jacksoni*) populations separated by similar geographic distances, (e.g., Palos Verdes vs. Monterey Bay, *F*st = 0.71; and Palos Verdes vs. Tomales Bay, *F*st = 0.74). Finally, we evaluated the most likely number of clusters in the combined data set, and found that one and two discrete clusters was equivocal (with five of 10 runs indicating a single cluster), using a maximum-likelihood framework implemented in STRUCTURE 2.3.2 (2000). Therefore, our STRUCTURE analyses could not distinguish between a single panmictic, or two distinct populations. We conclude that in this case, differences in reproductive strategies between central and southern California *C. aggregata* populations are attributed to differences in life history characteristics of the individuals sampled such as standard length and age, rather than population level differences in the genetic basis of reproductive tactics.

### Uterine complexity and spatial patterns of paternity

**Figure 8:**
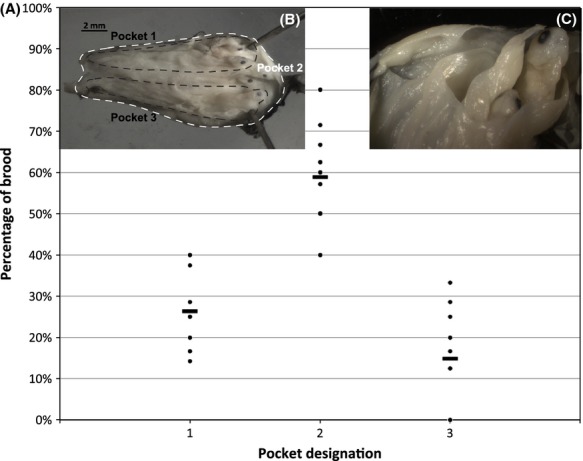
(A) Distribution of larvae within ovaries of *Cymatogaster aggregata*, *n* = 10. (B) Dissected uterus of *C. aggregata*. Dorsal view of opened uterine lumen, anterior to the left. pockets 1, 2 and 3 are outlined. Pocket 2 encompasses everything inside the uterus that is not pocket 1 or 3. Note the eyes of larvae, mostly oriented posteriorly. (C) Close up of surfperch larvae extruding from pocket 1 (anterior to the left).

The epithelial sheets within the uterus form three nearly separate compartments in *C. aggregata*: two smaller side pockets that open posteriorly and a third larger space, which encompasses the remaining lumen (Fig. 8). Each pocket has an epithelial sheet that loosely divides it in half; similar to what has been reported for two different embiotocine species (Behrens 1977).

**Figure 9:**
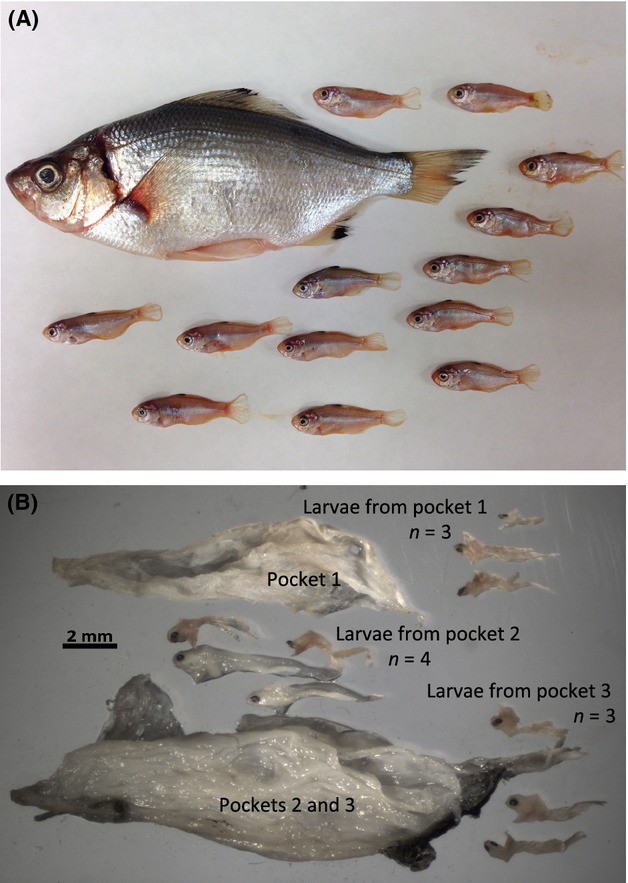
Pregnant female spotfin surfperch and offspring (A) illustrating the typical condition with little variation in developmental stage of offspring and (B) one female with superfetation (i.e., some embryos that appear to be developing normally while others are atrophied or underdeveloped).

Liu and Avise (2011) observed paternal skew in 31% of the southern California *C. aggregata* broods and Reisser et al. (2009) reported skew in 42% of both *Embiotoca jacksoni* and *E. lateralis* broods, suggesting that some form of sperm competition and/or cryptic female choice plays a role in determining paternity in approximately a third of these broods. It is possible that the complexity of the uterus may be conducive to cryptic female choice through selective sperm storage, fertilization or embryonic nutrition, and mitigation of resources to specific folds in the uterine sac. Postcopulatory mate selection occurs in other fishes with significant parental care, including the sex-role reversed male Gulf pipefish, which reabsorbs eggs from less desirable females to retain resources for future broods (Paczolt and Jones 2010). We evaluated spatial patterns of paternity between the pockets in 10 female *C. aggregata*. However, reproductive skew in paternal contribution was observed in only two of the 20 *C. aggregata* families (9.5%) with multiple paternity and in three of 24 *H. anale* families (12.5%). Further, of the uterine sacs that were preserved intact, we observed no obvious spatial patterns of paternity. While the potential for cryptic female choice and paternal skew may be high in surfperches due to their unique reproductive cycle/anatomy, our study did not have the power to detect skew due to the small brood sizes associated with the smaller female standard lengths in *C. aggregata* and limited variation in microsatellite loci in *H. anale*. However, our analysis did reveal an unexpected pattern of larval distribution between the two smaller pockets. Pockets 1 and 3 are approximately the same size (Fig. 8), yet a higher proportion of the brood was found in pocket 1 than pocket 3 in six of the 10 families (*x* = 26%, 15%, for pockets 1, 3, respectively), which was nearly significant (Mann–Whitney *U*-test, *N*_1_= *N*_2_ = 10, *U* = 88.5, *P* = 0.052). Further, while developmental stage was nearly uniform within broods, we sampled one female whose brood displayed atypical variation in size and developmental stage (Fig. 9). This is the first documentation of superfetation in surfperches, and when combined with the uneven distribution of embryos between pockets is suggestive of complex patterns of resource and/or paternity allocation.

## Discussion

Because only derived Embiotocid species have been characterized with respect to multiple paternity, it was previously unclear whether multiple paternity is a reproductive tactic shared by all members of the surfperch family. The family is divided into two monophyletic subfamilies: the Embiotocines, which have soft flask organs (penis-like structures) and occur primarily in stable, demersal habitats with structure, such as kelp or rocky outcrops; and the Amphisticines, which lack an analogous flask organ but exhibit anal fins with hook-like structures and occur primarily in habitats with more wave energy, over sandy shores near surf zones. We evaluated the basal Amphisticine taxon, *Hyperprosopon anale* and found that this species does exhibit multiple paternity as a reproductive strategy. Therefore, it is apparent that sperm storage and polyandry are common strategies in both the Amphistichinae and Embiotocinae, and likely arose in the common ancestor of the family.

Evaluating the subtleties in patterns of paternity, and the potential for a female Bateman gradient in *Hypersprosopon anale* was problematic for two reasons. First, the number of families sampled was moderate (*n* = 24), even though the samples spanned a greater range of female standard lengths, representing more reproductive life-history stages than either study on shiner surfperch (maximum SL reported for spotfin = 6 inches, shiner = 7–8 inches; Miller and Lea 1972). As such, analysis of the total data set suggests that a female Bateman gradient may occur in spotfin surfperch (Table 3, Fig. 5). However, the relationship is no longer significant after correcting for differences in brood size, and therefore, it is clear from this and previous studies that sample sizes must be significantly larger to detect a Bateman gradient when present. Second, two of the three microsatellite loci that successfully amplified for this species exhibited limited variability; therefore, future studies would benefit from genomic scale variation, such as RADTags, to increase the accuracy in paternity assignments. This would also increase the power to characterize differences in mating strategies (i.e., slope of Bateman gradients) and reproductive skew between embiotocid species and subfamilies. Because Amphisticines exhibit differences in anal fin morphology that may have evolved in association with a more dynamic habitat, it is possible that mating strategies and sexual selection operate differently between these two Embiotocid clades. If there is variation in Bateman gradients among the Embiotocids, we might expect to see stronger sexual selection by females in those species where the relationship between female size and number of offspring is strong, yet the relationship between number of mates and number of offspring (female Bateman gradient) is weak. In other words, if Bateman gradients vary with reproductive strategy and habitat type, this could explain some of the contentiousness in the literature over this evolutionary theory on sexual selection.

Surfperches exhibit one of the most derived reproductive strategies among vertebrates. By giving birth to live young that are sexually mature in some cases (Schultz 2008), they essentially give birth to teenagers, representing a disproportionate investment in offspring survivorship by females relative to males. Because surfperches have no paternal care, and there is no evidence for monogamy or extreme bias in sex ratio, this system is likely nonresource based. Previous studies have detected a positive correlation between standard length and brood size, a common observation among fishes, but no significant relationship between brood size and number of sires. Interestingly, we found that these studies, the current study included, sampled a limited size range of females. When we combined our data on *Cymatogaster aggregata* with data from a previous study, spanning a broader range of female standard lengths, we were able to infer a positive correlation between brood size and number of sires, suggesting increasing fertility and a change in reproductive tactic with respect to the number of sires throughout the reproductive life history of females (Fig. 9). This correlation is referred to as a Bateman gradient and is expected in males but not females (Bateman 1948; Arnold and Duvall 1994; Jones et al. 2005). While female Bateman gradients have been observed in some taxa, including four fishes (see Gerlach et al. 2012). This is the first time a Bateman gradient has been inferred for any Embiotocid species. This implies that sexual selection may be strong in both sexes, where males prefer larger females and females may exhibit cryptic choice. Taken together, sexual selection in this system is likely to be complex with the potential for conflicting optima. For example, male guppies prefer larger females but will accept smaller females under threat of sperm competition and multiple paternity (Jeswiet 2012), while female guppies gain several benefits from multiple mating including shorter gestation times, larger broods, and offspring with better schooling abilities and escape responses than their singly mated counterparts (Evans 2000). Other benefits of polyandry include bet-hedging, increased genetic diversity within broods, mitigation of maternal-fetus conflict (Zeh and Zeh 1996, 1997, 2001), nuptial gifts (Arnold and Duvall 1994) and the potential for both sperm competition and female cryptic choice (e.g., postcopulatory resource or sperm allocation by the female, Keller et al. 1995; Yasui 2001). Bet hedging can benefit females by mitigating errors in the assessment of male fitness and is most likely to play a role in small populations (Yasui 2001). Increased genetic diversity within broods may increase female fitness in variable or fluctuating environmental conditions (Yasui 1998, 2001). Because surfperch embryos derive nutrition in utero, it is possible that stored sperm provides some benefit to the developing offspring. Sperm competition between two or more males contributes to genetic robustness within broods, enhancing both female fitness and offspring viability (Keller et al. 1995). It may also increase the probability of “sexy sons” that produce more competitive sperm (Curtsinger 1991). Sexual selection by females increases the potential for acquiring “good genes” and/or “sexy sons” if loci associated with these traits are linked to indicator traits that are recognized by the female. Surfperches exhibit complex reproductive behaviors (Wiebe 1968) and sexual dimorphism in anal fin coloration and secondary sex traits (Westphal et al. 2011) that are likely apparent to females. Remarkably, female surfperches have a protracted reproductive cycle lasting all year, Figure 1, which is amenable to both cryptic and direct female choice across multiple stages, including mate selection(s), prolonged sperm storage, sperm competition, protracted oogenesis, and extensive gestation with maternal nutrition. Therefore, polyandry may impose a number of potential benefits to females that warrants further investigation, particularly in cases where a female Bateman gradient exists.

Several aspects of surfperch reproductive life history suggest the potential for female cryptic choice. First, females continue to produce oocytes well after mating, when male gonadosomatic index drops and mature sperm are no longer present in males (Froeshke et al. 2007). This implies that the number of mates acquired by a female may be a factor in the number of offspring, consistent with a true Bateman gradient. Second, we describe the uterine structure in *C. aggregata,* as epithelial sheets forming three distinct compartments with internal complexity similar to *Embiotoca* (Reisser et al. 2009). Although our data lacked the power to detect significance in pocket allocation and paternal skew, skew was detected in *C. aggregata* by Liu and Avise (2011) and *Embiotoca* (Reisser et al. 2009). Skewed paternity combined with uterine spatial complexity associated with the distribution of juveniles could indicate sperm competition and/or postcopulatory mate selection. Because females store sperm for up to 5 months and gestation spans for an additional 6 months with embryonic nutrition supplied by the mother, female surfperches may have more control over paternity and offspring viability than most fishes. Finally, we found evidence for maternal influence on offspring viability in one female *C. aggregata* with early developing embryos that exhibited unusual variation in development, (i.e., superfetation, Fig. 9), while mature juveniles, from all other families sampled, exhibit low variability in standard length within, (1–2 mm) and developmental stage before birth.
